# A Novel Single-Stage Procedure for Increasing the Width of Attached Gingiva and Eliminating the Aberrant Frenal Attachment

**Published:** 2015-03

**Authors:** Santhosh Kumar, Gautham Suresh P, K. Meena Anand

**Affiliations:** a Dept. of Periodontology Manipal College of Dental Sciences, Manipal University, Manipal - 576104 Udupi Dist, Karnataka, India.; b Dept. of Dental surgery PSG, Institute of Medical Sciences (PSGIMSR) Peelamedu Coimbatore 641004, India.

**Keywords:** Gingival recession, Frenum, Attached gingival, Vertical incision, Horizontal incision

## Abstract

Common treatment for buccal gingival recession caused by an aberrant frenal attachment includes elimination of the frenum and treatment of the gingival recession by soft tissue graft to increase the width of the attached gingiva that in turn results in root coverage. Keratinised gingival, if present in adequate amount, maintains the gingival health by protecting the marginal gingiva. This not only considers the desires of the patient but also explores the potential regenerative capacity of the tissues. This report describes a novel single-stage procedure for increasing the width of the attached gingiva and eliminating the aberrant frenal attachment.

## Introduction

The width of the attached gingiva varies in different individuals and on different teeth of the same individual. [1] However, adequate amount of keratinised gingiva maintains gingival health by protecting the marginal gingiva by preventing the progression of gingival recession. [2-3] Teeth with subgingival restoration and a narrow zone or absence of attached gingiva have higher inflammation than those with wider zone of attached gingival. [4]

Buccal gingival recession defects have been treated by using various procedures [5] with good and predictable outcomes. Buccal gingival recession due to high frenal attachment requires two-stage procedures to be treated. This report describes a single-stage procedure to eliminate the frenum in addition to increase the width of attached gingiva.

## Case report

A healthy 28-year old male patient referred to the Department of Periodontics, Manipal College of Dental Sciences, complaining of bleeding gums while brushing and abrasion in relation to the upper right back tooth.

**Figure 1:**
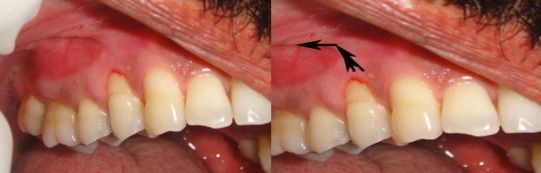
Aberrant frenal attachment

On examination, the patient had poor oral hygiene and there was gingival abrasion and localised Miller’s [6] class 2 recession extending beyond the mucogingival junction due to the aberrant buccal frenal attachment in relation to tooth number 14 (Figure 1). The patient was treated by a single-stage procedure which made use of new technique of folding the apical connective tissue over the defect to increase the width of the attached gingiva and eliminate the aberrant frenum. Three months prior to the surgical procedure, the patient was asked to maintain adequate plaque control by a vertical method of brushing technique. Later, the patient was taught to use modified Stillman’s method of brushing on the specific areas of gingival recession.

**Figure 2:**
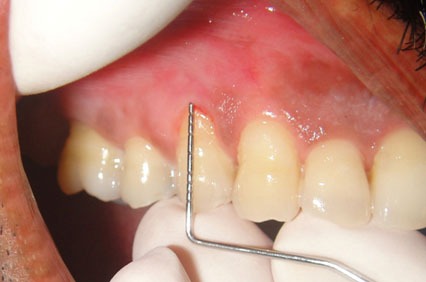
Gingival recession measurement by using a probe

The procedure was planned and after obtaining consent the patient was enrolled for the technique. Local anaesthetic injection 1:200000 Lidocaine hydrochloride with adrenaline (Xylocaine 2% Adrenaline, Astra Zeneca Pharma) was used by local infiltration and the area was freed from saliva. The recession was measured using the periodontal probe with William’s markings (Figure 2).

**Figure 3:**
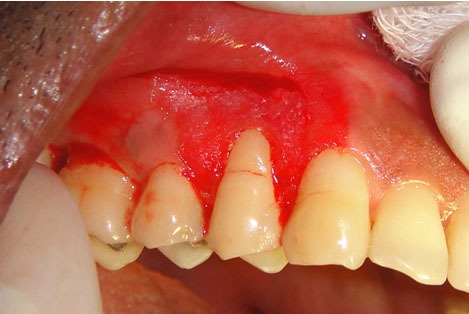
De-epithelized tissue showing exposed connective tissue

The surface epithelium was denuded using a slow-speed hand piece. The area of de-epithelisation was extended till and beyond 2–3mm of the adjacent tooth line angle around the area of recession (Figure 3). Deepithelialisation was carried out beyond the mucogingival junction, the papillae was also denuded.

**Figure 4:**
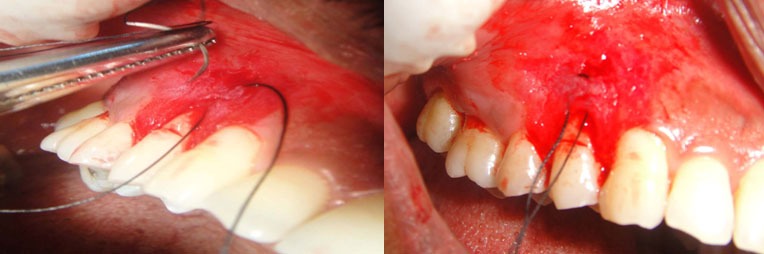
Suture passed through the sulcus

Sulcular incision was extended till the apical extent; Silk suture 4-0 (Mersilk; Ethicon, Johnson & Johnson) was inserted through the sulcus and bite is taken 1-2 mm coronal to the probable horizontal incision. The needle was removed from the external surface then a bite was taken from the external surface of the gingiva parallel to the first bite and then it was passed along the tooth and removed through the sulcus (Figure 4).

**Figure 5:**
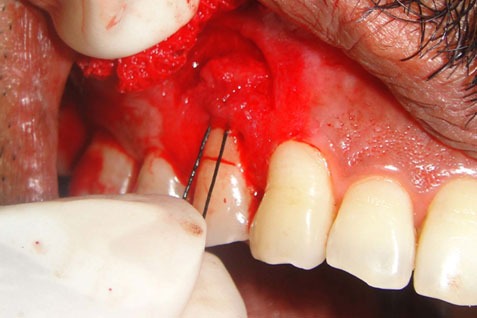
Excision of the connective tissue from the apical area

Vertical incisions were placed at the line angle of the adjacent tooth, converging towards the apex about one quarter more than the height of the apparent recession. Horizontal incision was placed at the bottom of the vertical incision to eliminate the frenum. The connective tissue was reflected from apical area using sharp dissection (Figure 5).

**Figure 6:**
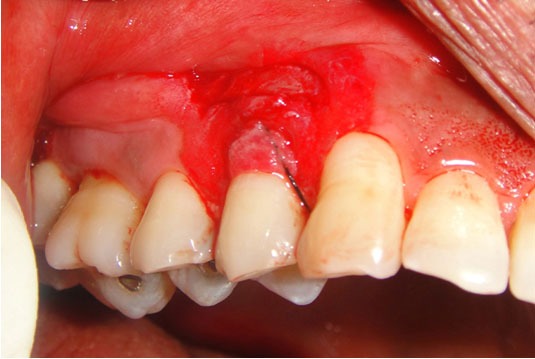
Connective tissue folded and sutured

The connective tissue was pulled through the sulcus and adapted over the recession using the suture and knotted on the palatal surface (Figure 6).

The flap was still intact and attached to the adjacent attached gingiva.

**Figure 7:**
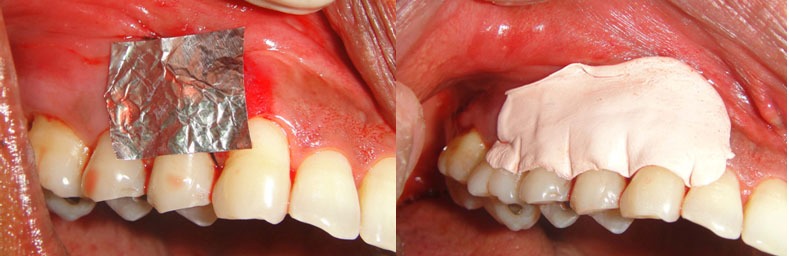
Periodontal dressing in place

Tin foil was placed; periodontal dressing (Coe-Pak, GC America) was used to cover this area to secure and protect the graft (Figure 7).

**Figure 8:**
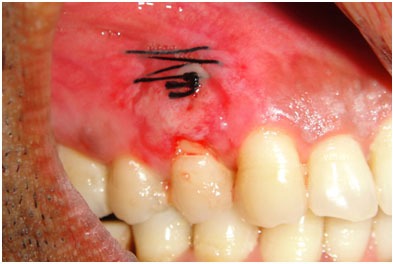
Recall after 15 days for suture removal

Analgesic was prescribed for 3 days and the patient was asked to rinse with 0.2% chlorhexidine mouthwash (Hexidine; ICPA Health Products) for 15 days. After fifteen days of the treatment procedure, the periodontal status was improved (Figure 8).

**Figure 9:**
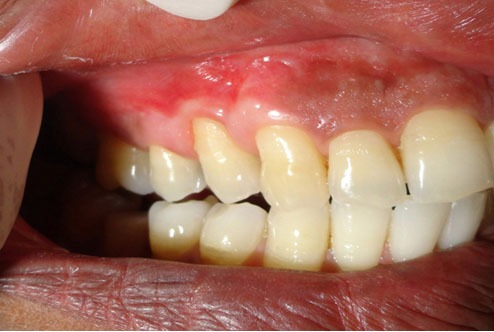
One-year recall

One year recall period showed increase in the width of the attached gingiva to 2-3mm; there was an increase in the width of the attached gingiva on the canine area and also there was improvement in the patient’s overall oral hygiene status (Figure 9).

## Discussion

Lang and Löe had suggested that keratinized gingiva less than 2.0 mm (corresponding to <1.0 mm of attached gingiva) facilitate the introduction of plaque into the gingival crevice by a movable gingival margin. It would probably present a clinical problem by making it difficult to remove by conventional tooth brushing. [4] Investigations by Miyasota *et al.*, Tenenbaum and Hangorsky and Bissada disapproved this concept but no mention was made in these studies about the restorations close to the gingiva inhibiting the plaque removal in the zones of keratinized gingiva less than 2.0mm. [7]

Buccal frenum can be corrected by simple frenotomy and then the denuded area can be covered using a free gingival graft. After relieving the frenum the recession is covered by any of the pedicle graft, [8] the free soft tissue graft, [9] the acellular dermal matrix [10] or the modified apically repositioned flap to increase the dimension of attached gingiva. [11]

The advantage of the connective tissue graft is complete root coverage, but it requires two sites for the procedure. It can also produce anaesthetic scars. The coronally positioned graft is not advisable in areas of frenal pull. Cost factor and time can hinder the acellular dermal matrix utilisation. Using this procedure, the root coverage potential of the folded connective tissue can be explored in future researches. 

## Conclusion

This novel and much simpler technique to increase the width of attached gingiva and consecutively increase the keratinized gingiva can prevent frequent appointments and decrease the procedure duration. Moreover, it does not include the palatal donor site; thus it is considered to be less traumatic. The factors which could affect the success of the procedure include aberrant buccal frenal attachment with Miller’s class 3 and class 4 gingival recessions. Using larger sample size and longer duration will help in determining the success and predictability of the procedure. 
